# P-480. Oral vancomycin prophylaxis to prevent recurrence of Clostridioides difficile infection in children with cancer

**DOI:** 10.1093/ofid/ofaf695.695

**Published:** 2026-01-11

**Authors:** Isabela (Bela) DeJohn, Chunyan Liu, Maryam Mysorewala, Justin Markham, Joshua D Courter, Hilary Miller-Handley, Mark Murphy, Grant C Paulsen, Lara A Danziger-Isakov, William R Otto

**Affiliations:** University of Cincinnati College of Medicine, Cincinnati, OH; Cincinnati Children's Hospital, Cincinnati, Ohio; Cincinnati Children's Hospital Medical Center, Cincinnati, Ohio; Cincinnati Children's Hospital Medical Center, Cincinnati, Ohio; Cincinnati Children's Hospital Medical Center, Cincinnati, Ohio; Cincinnati Children's Hospital Medical Center, Cincinnati, Ohio; Cincinnati Children's Hospital Medical Center, Cincinnati, Ohio; Cincinnati Children's Hospital Medical Center, Cincinnati, Ohio; Cincinnati Children's Hospital, Cincinnati, Ohio; Cincinnati Children's Hospital Medical Center, Cincinnati, Ohio

## Abstract

**Background:**

Recurrent *Clostridioides difficile* infection (CDI) is a significant burden for children with cancer. Studies in adults reported that oral vancomycin prophylaxis (OVP) decreased risk of recurrent CDI (rCDI); pediatric data is limited. We evaluated the impact of secondary OVP during broad-spectrum antibiotic (BSA) administration on rCDI in children with cancer.

**Table 1:**
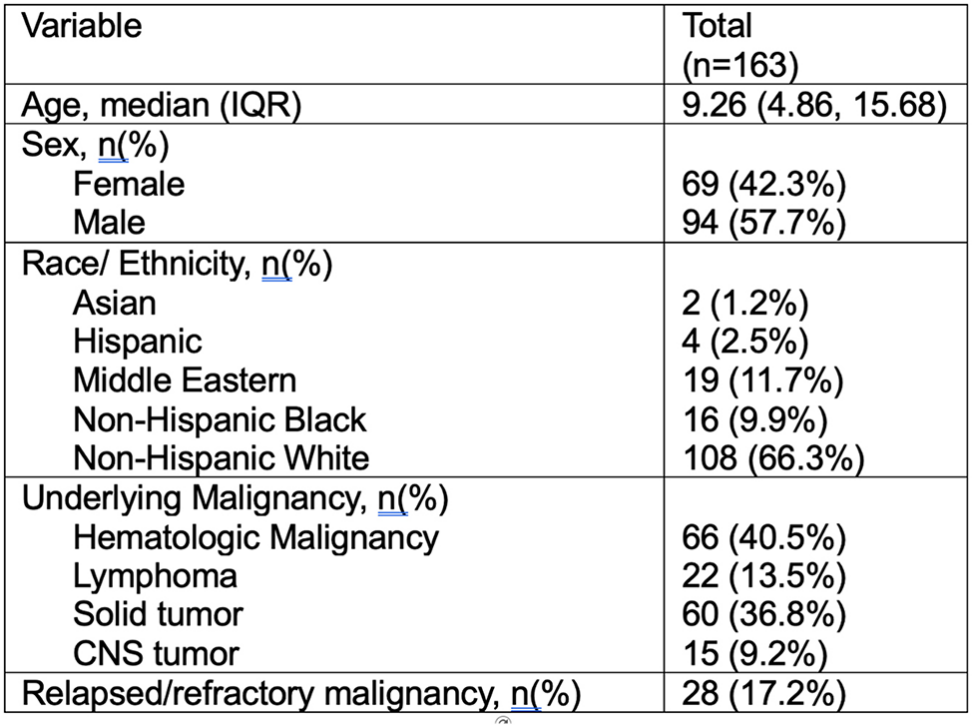
Demographic and clinical information for the cohort

**Table 2:**
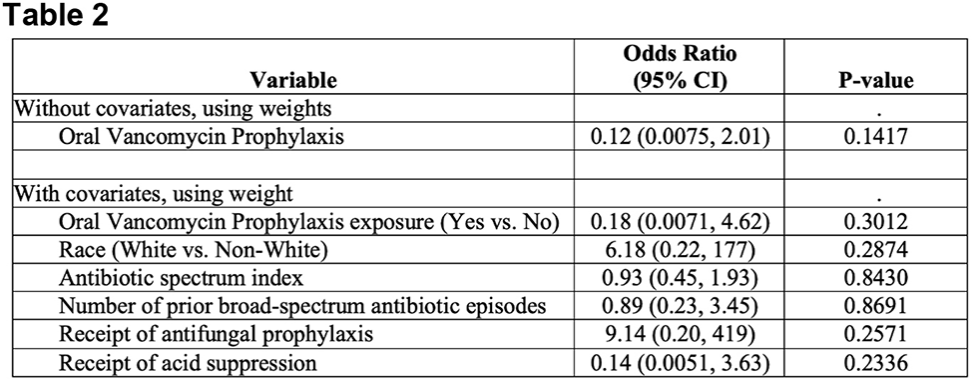
Impact of oral vancomycin prophylaxis on recurrence of C. difficile colitis during broad spectrum antibiotic use

**Methods:**

This was a retrospective study of children with cancer and their first CDI episode. Data related to CDI episodes were collected, including use of OVP during subsequent courses of BSA. The incidence of rCDI within 6 months of first CDI was calculated.

Propensity score (PS) analyses were performed to determine the impact of OVP in preventing rCDI. Cases were matched to controls using a combination of caliper matching on PS and exact matching. For patients with multiple episodes of BSA use, the “no prophylaxis” episodes served as the control for OVP episodes. Patients who only received OVP were matched to control patients who did not receive OVP. A weighted logistic regression model was used for the outcome analysis.

**Results:**

A total of 163 children with CDI were included; the cohort was predominately White and male (Table 1). Severe CDI occurred in 16/163 (9.82%) of episodes, while severe complicated CDI occurred in 19/163 (12.03%). The most common treatment regimen for first CDI episodes was oral vancomycin (102/163, 62.58%), followed by metronidazole (19/163, 11.66%). Excluding patients lost to follow-up, 44/155 (28.39%) of children had rCDI. Severe CDI occurred in 1/44 (2.27%) of rCDI episodes, while severe complicated CDI occurred in 6/44 (13.64%). A vancomycin taper was the most common treatment for rCDI (17/44, 38.64%) followed by fidaxomicin 11/44 (25%). The PS analysis was performed on a subset of patients (n=60). Outcome analysis on the matched data did not show significant association between OVP and reduced incidence of rCDI during BSA use (Table 2).

**Conclusion:**

In this retrospective cohort study of pediatric oncology patients with their first CDI episode, rCDI was common. Preliminary PS analyses suggest that use of OVP during episodes of BSA was not associated with reduction in rCDI, though updated analyses are pending.

**Disclosures:**

Grant C. Paulsen, MD, Moderna, Inc: Grant/Research Support|Pfizer: Grant/Research Support|Sanofi: Grant/Research Support Lara A. Danziger-Isakov, MD, MPH, Aicuris: Grant/Research Support|Ansun BioPharma: Grant/Research Support|Astellas: Advisor/Consultant|Astellas: Grant/Research Support|Merck: Advisor/Consultant|Merck: Grant/Research Support|Pfizer (Any division): Grant/Research Support|Takeda: Grant/Research Support

